# Is medical urgency of elderly patients with traumatic brain injury underestimated by emergency department triage?

**DOI:** 10.1080/03009734.2019.1706674

**Published:** 2020-01-28

**Authors:** Toralph Ruge, Axel C. Carlsson, Magnus Hellstrom, Per Wihlborg, Johan Undén

**Affiliations:** a Department of Clinical Sciences Malmö, University Hospital of Skåne, Sweden;; b Department of Emergency Medicine, Karolinska University Hospital, Huddinge, Stockholm, Sweden;; c Division of Family Medicine and Primary Care, Department of Neurobiology, Care Sciences and Society, Karolinska Institutet, Huddinge, Sweden;; d Department of Medical Sciences, Cardiovascular Epidemiology, Uppsala University, Uppsala, Sweden;; e Department of Surgical and Perioperative Science, Umeå University, Umeå, Sweden;; f Department of Operation and Intensive Care, Halmstad Hospital, Halmstad, Sweden;; g Anaesthesiology and Intensive Care Medicine, Lund University, Lund, Sweden

**Keywords:** Elderly patients, emergency department, RETTS-A, traumatic brain injury

## Abstract

**Background:** Mortality is high among elderly patients with traumatic brain injury (TBI). Recent data suggest that early surgical intervention and aggressive rehabilitation may reduce mortality rates even in elderly patients. Our aim was therefore to study the Rapid Emergency Triage and Treatment System–Adult (RETTS-A) triage of patients with isolated TBI and examine the differences in acute management according to age.

**Methods:** We included 306 adult patients with isolated severe TBI and an abbreviated injury scale (AIS) score ≥3. Using a cut-off of 60 years of age, differences in triage priority according to RETTS-A, time to first computed tomography (CT) scan, length of hospital stay (LOS), and 30-day survival were studied.

**Results:** In patients with an AIS score of 3 and 4, we observed that elderly patients had a longer time from admission to first CT scan. In addition, we observed that elderly patients were less often triaged with the highest priority level, despite similar AIS scores. LOS was significantly higher in elderly patients (median 9 days compared with 3 days for younger patients, *p* < 0.001). Finally, age, triage priority, and AIS score were independent risk factors for mortality.

**Conclusion:** Elderly patients with isolated TBI are managed differently than younger patients, which could be due to an under-triage of elderly patients by RETTS-A.

## Introduction

Traumatic brain injury (TBI) is one of the leading causes of death in the Western world, with an incidence between 47 and 849 per 100,000 habitants per year (1,2). Elderly patients are particularly vulnerable and are overrepresented among patients with TBI. They also suffer from a higher mortality, prolonged hospitalisation, and significant long-term disability after TBI (2). The main cause of TBI among elderly patients is standing falls, followed by road traffic accidents (3).

Early identification of severe intracranial injury, such as those needing specific intervention, is important in order to improve outcome. Encouraging data from several studies indicate that early surgical treatment and aggressive rehabilitation also improve outcome after TBI in elderly patients (4). Therefore, appropriate triage of elderly patients with TBI is critical in order to identify patients who would benefit from early surgical intervention. Rapid Emergency Triage and Treatment System–Adult (RETTS-A) is the most common triage system used in Sweden. It uses a combination of the patient’s presenting symptoms and signs, in addition to vital sign values, to determine triage priority. The RETTS-A triage scale levels are classed as red, orange, yellow, green, and blue, in declining level of acuteness (5).

Current literature indicates under-triage of elderly trauma patients, including those with TBI (6). Although an altered level of consciousness is associated with increased mortality (7), most elderly patients have relatively subtle changes of level of consciousness compared with younger patients, despite potentially serious intracranial complications, which may increase the risk of under-triage (8).

Some of the data presented here were published previously in a smaller pilot study with the main aim to study pre-hospital care of patients with TBI (9). The main finding was that elderly patients had higher mortality and had to wait longer for diagnostic imaging, compared to younger patients. The effect of age on triage priority according to RETTS-A in patients with isolated TBI has not been studied. Our aim was therefore to study the RETTS-A triage of patients with isolated TBI and examine the differences in acute management according to age.

## Materials and methods

### Study design

This was a retrospective cohort study.

### Study setting

Patients were included from two counties in Sweden: Västerbotten between 2011 and 2012 (Lycksele, Skellefteå, and Umeå), and Skåne (University Hospitals in Malmö and Lund) between 2013 and 2014. Patients from Västerbotten were identified through the Injury Data Base (IDB; www.socialstyrelsen.se), and patients from Skåne were identified through the Patients Administrative System (PAS; patient medical record). Variables were documented according to the SweTrau (Swedish national trauma register; http://rcsyd.se/swetrau) manual and were subsequently retrieved from the IDB and PAS.

### Ethical considerations

The study was approved by the regional Ethical Review Board in Stockholm (2014/1822–31/4).

### Study population

Inclusion criteria were all patients with an isolated TBI (i.e., no other traumatic injuries), ICD 10 codes S06.1–S06.9 or S02.0–S02.1 (S06.0, concussion; S06.1, traumatic cerebral oedema; S06.2, diffuse TBI; S06.3, focal TBI; S06.4, epidural haemorrhage; S06.5, traumatic subdural haemorrhage; S06.6, traumatic subarachnoid haemorrhage; S06.8, other specified intracranial injuries; S06.9, unspecified intracranial injury; S02.0, fracture of vault of skull; and S02.1, fracture of base of skull) and an abbreviated injury scale (AIS; AIS version 2005 Update 2008, https://www.aaam.org/abbreviated-injury-scale-ais/) score ≥3 (10). Exclusion criteria were patients with multiple injuries and patients with missing data. For the purpose of the study, patients were divided into those below the age of 60 and those 60 years or older (defined as elderly in this study).

### Data collection

The collected variables included age, sex, cause of injury, use of anticoagulants (patients were asked for the use of either warfarin, NOAK [new oral anticoagulants], or acetylsalicylic acid), triage priority according to RETTS-A (5), time to first computed tomography (CT) scan (defined as time from emergency department [ED] admission [arrival] to CT), total length of hospital stay (LOS; measured in number of days from ED arrival to discharge from in-hospital care, only including patients surviving 30 days), and 30-day mortality (counted from ED arrival). Rapid Emergency Triage and Treatment System–Adult (RETTS-A) was introduced in Sweden in 2005 and is now the most common triage tool in Sweden and Scandinavia, including also pre-hospital care. RETTS-A uses a combination of the patient’s presenting symptoms (chief complaint) and the patient’s vital signs in order to determine triage priority. This results in one RETTS-A triage priority based on the single most deviating vital sign and one based on the severity of the emergency signs and symptoms (ESS). The more urgent of the two becomes the patient’s final triage priority. The RETTS-A triage scale levels are: red, orange, yellow, green, and blue, in declining level of acuteness. Vital signs include heartbeat (rate, regular/unregular rhythm), respiratory rate, level of consciousness, and body temperature. Medical urgency increases if patient values deviate from the normal range (7). Triage was performed by trained nurses at each centre. The lowest three priority levels (level blue, green, and yellow) were combined and redefined as priority level I (not life-threatening), while clinical priority level II corresponded to RETTS-A triage level orange (possibly life-threatening), and clinical priority level III corresponded to RETTS-A level triage red (life-threatening). The AIS scale relies on the anatomical location and severity of injuries. The purpose of the scale is to describe how life-threatening the anatomical specific injury is as such for the patient, but it does not include an overall evaluation of the patient. The AIS scoring system classifies an individual injury by body region according to its relative severity on a 6-point scale (1 = minor, and 6 = maximal).

### Outcomes

The primary outcome was triage priority according to RETTS-A. The secondary outcome measures were time from ED admission to first CT scan, total length of hospital stay (LOS), and 30-day mortality.

### Statistical analysis

Data were analysed using SPSS software, version 18.0. Continuous variables at baseline were not normally distributed; therefore, median values and interquartile range were calculated. Non-parametric statistical tests were used for univariate analysis, Mann–Whitney *U*-test was used to compare differences between two groups, and Kruskal–Wallis test was used where more than two groups were included into the analyses. The Pearson chi-square test and Fisher’s exact test were applied when the independent variable was categorical.

Univariate and multiple linear regression analyses were used to determine the association between the response variable LOS (days) and patient demographics and clinical factors among patients surviving until hospital discharge. Hospital LOS was highly right-skewed and was therefore transformed and analysed using the natural logarithmic scale in the multiple linear regression in order to obtain the residuals from the normally distributed model.

Binary logistic regression modelling with stepwise variable selection was performed to identify predictors (Tables 3 and 4) of death during in-hospital stay. All tests were two-sided, and *p* values <0.05 were considered statistically significant.

### Availability of data and material

The datasets used and/or analysed in the present study are available from the corresponding author upon request.

## Results

### Patient characteristics

A total of 330 patients were initially recruited. Of these, 24 patients were excluded due to missing data, leaving 306 included patients. Patients were divided into two subgroups: those below the age of 60 years (*n* = 87) and those ≥60 years (*n* = 219). There were more men in both age groups, with a higher proportion in the <60 years group (Table 1). AIS scores were similarly distributed among both groups. The main mechanisms of injury for patients below the age of 60 were standing falls (including falls from <1 to ≥1 m, 30%) and blunt trauma (20%). For patients 60 years or older, the main mechanism of injury was falls from the same or different levels (70%). The use of anticoagulant drugs (warfarin and acetylsalicylic acid) was more common in patients 60 years or older compared to patients below the age of 60.

**Table 1.** Patient characteristics.

Variable	Age < 60 years	Age ≥ 60 years	*p* Value
	(*n* = 87)	(*n* = 219)	
Age (years), median (IQR)	46 (26–56)	81 (72–86)	<0.001
Male, *n* (%)	62 (71.3)	124 (56.6)	0.012
AIS score, *n* (%)			0.314
3	52 (59.8)	112 (51.1)	
4	22 (25.3)	60 (27.4)	
5	13 (14.9)	47 (21.5)	
Triage priority, *n* (%)			0.013
Level I	30 (34.5)	94 (42.9)	
Level II	28 (32.2)	86 (39.3)	
Level III	29 (33.3)	39 (17.8)	
Anticoagulants, *n*(%)			<0.001
None	80 (94.1)	112 (51.6)	
Warfarin	2 (2.4)	44 (20.3)	
Acetylsalicylic acid	3 (3.5)	61 (28.1)	
Hospital, *n* (%)			0.214
Skåne	49 (56.3)	106 (48.4)	
Umeå	30 (34.5)	72 (73.0)	
Lycksele	4 (4.6)	17 (7.8)	
Skellefteå	4 (4.6)	24 (11.0)	
Time to first CT scan (min), median (IQR)	80 (45–131)	121 (66–227)	<0.001
Length of stay (days), median (IQR)	3 (1–10)	9 (3–18)	<0.001

Values are expressed as median (IQR), or median (%). Associations were tested using chi-square or Mann–Whitney *U*-test. *n* = 306 for all variables.

AIS: Abbreviated Injury Scale; IQR: interquartile range; Skåne: university hospitals in Malmö and Lund.

### Triage

We observed that a greater proportion of patients below the age of 60 were categorised with triage priority III compared with patients 60 years or older (Table 2). Among patients with AIS = 4 and 5, fewer patients below the age of 60 were triaged with triage priority I compared with patients ≥60 years (Table 2).

**Table 2.** Comparison of triage priority, mortality, time of management, and length of stay by AIS score.

	Patients < 60 years		Patients ≥ 60 years		*p* Value
Triage priority by AIS score (*n* = 306)	*n*		*n*		
AIS = 3, *n* (%)					
Level I		26 (50.0)		59 (52.7)	0.349
Level II		11 (21.2)		35 (31.2)	
Level III		15 (28.8)		18 (16.1)	
AIS = 4, *n* (%)					
Level I		2 (9.1)		17 (23.2)	0.022
Level II		11 (50.0)		31 (51.2)	
Level III		9 (40.9)		12 (25.6)	
AIS = 5, *n* (%)					
Level I		2 (15.4)		18 (38.3)	0.073
Level II		6 (46.2)		20 (42.6)	
Level III		5 (38.5)		9 (17.8)	
Mortality by AIS score (*n* = 306), *n* (%)					
AIS = 3		1 (1.9)		17 (15.2)	0.013^a^
AIS = 4		2 (9.1)		12 (20.0)	0.207^a^
AIS = 5		3 (23.1)		13 (27.7)	0.332^a^
Time to first CT scan by AIS score (*n* = 306; min), median (IQR)					
AIS = 3	52	94.0 (53.3–144.5)	112	138.0 (75.3–245.3)	0.005
AIS = 4	22	52.5 (38.8 –96.3)	60	113.0 (63.8–196.8)	0.002
AIS = 5	13	101.0 (58.5–172.5)	47	108.0 (47.0–214.0)	0.713
Hospital length of stay by AIS score (*n* = 258; days), median (IQR)					
AIS = 3	51	2.0 (1.0–4.5)	95	8.0 (3.0–14.0)	<0.001
AIS = 4	20	7.0 (1.5–11.8)	48	9.0 (3.0–17.8)	0.381
AIS = 5	10	8.0 (4.8–20.5)	34	12.0 (5.0–25.5)	0.509

Values are expressed as number (%), or median (IQR). Associations were tested using chi-square or Mann–Whitney *U-*test. For length of stay and time to first CT, analyses were only performed for patients who survived.

^a^Fisher’s exact test.

AIS: Abbreviated Injury Scale (Mann–Whitney *U*-test).

### Time from admission to first CT scan

The time from ED admission to first CT scan was shorter for patients below the age of 60 and with AIS scores of 3 and 4 compared with patients 60 years or older (*p* < 0.01, Table 2). There was no significant difference between the two age categories with respect to time from admission to first CT scan for AIS scores 5. We found no association between time from ED admission to first CT scan and hospital LOS (Tables 3 and 4) or mortality (odds ratio = 1.00, 95% confidence interval 0.99–1.01, *p* = 0.374, data not shown in Table 5).

**Table 3.** Bivariate analysis of test independent variable association with LOS.

Variable	Test type	Test value	*p* value
Gender	Mann–Whitney	*U* = −0.74	0.46
Age	Spearman’s rho	*ρ* = 0.24	<0.001
AIS score	Kruskal–Wallis	*χ*^2^(2) = 4.30	0.04
Triage priority	Kruskal–Wallis	*χ*^2^(2) = 13.84	<0.001
Time to first CT scan	Spearman’s rho	*ρ* = −0.09	0.17
Anticoagulants	Kruskal–Wallis	*χ*^2^(2) = 2.87	0.24
Hospital	Kruskal–Wallis	*χ*^2^(3) = 9.58	0.02

**Table 4.** Independent factors associated with length of stay. Multiple linear regression on logarithmic transformed response variable length of stay (LOS).^a^

Variable	B (95% CI)	Standard error	*p* Value
Intercept	−0.22	0.36	
Age	0.02 (0.01–0.03)	0.004	<0.001
Gender			
Female	Reference		
Male	0.13 (−0.18–0.45)	0.16	0.41
Triage priority			
Level I	Reference		
Level II	0.32 (−0.04–0.69)	0.18	0.08
Level III	0.98 (0.52–1.45)	0.24	<0.001
Hospital			
Skåne	Reference		
Umeå	0.48 (0.14–0.83)	0.18	0.01
Lycksele	0.52 (−0.09–1.13)	0.31	0.09
Skellefteå	0.66 (0.08–1.24)	0.30	0.027
AIS score			
AIS = 3	Reference		
AIS = 4	0.22 (−0.13–0.56)	0.18	0.22
AIS = 5	0.81 (0.41–1.21)	0.20	<0.001
Anticoagulants			
None	Reference		
Warfarin	0.13 (−0.21–0.48)	0.18	0.46
Acetylsalicylic acid	0.06 (−0.25–0.38)	0.16	0.70
Time to first CT scan	0.00 (−0.01–0.01)	0.004	0.38

Model: *F*(10,213) = 6.08, *p* < 0.001, *R*^2^ = 22.2%.

^a^Respond variables (LOS) are logarithmic transform. Interpretation of *B*: %Δ*y* = 100⋅ *B* ⋅Δ*x* ‘if we change *x* by one unit, we expect our *y* variable to change by 100⋅*B* percent’ for small *B*, where *B*, unstandardised coefficients.

AIS: Abbreviated Injury Scale; 95% CI: 95% confidence interval; Skåne: university hospitals in Malmö and Lund.

**Table 5.** Mortality according to demographic and clinical factors.

	Died, *n*/total	Standard error	Odds ratio (95% CI)	Adjusted *p* value^a^
Total	48/306			
Age		0.01	1.05 (1.02–1.07)	<0.001
Gender				
Male	28/187		1	
Female	20/119	0.37	0.79 (0.39–1.62)	0.52
Triage priority				0.005
Level I	10/100		1	
Level II	15/106	0.46	1.18 (0.47–2.92)	0.73
Level III	19/62	0.50	4.62 (1.75–12.20)	0.002
Unknown	4/38	0.66	1.45 (0.39–5.30)	0.58
AIS score				0.042
AIS = 3	18/164		1	
AIS = 4	14/81	0.42	1.61 (0.71–3.64)	0.26
AIS = 5	16/61	0.42	2.78 (1.22–6.29)	0.014
Hospital (#)				0.41
Skåne	26/155		1	
Umeå	12/102	0.46	1.12 (0.46–2.78)	0.79
Lycksele	3/21	0.74	1.15 (0.27–4.92)	0.85
Skellefteå	7/28	0.61	2.82 (0.85–9.34)	0.09
Anticoagulants				0.38
None	29/192		1	
Warfarin	9/46	0.63	0.64 (0.19–2.20)	0.48
Acetylsalicylic acid	9/64	0.50	1.35 (0.50–3.69)	0.55
Time to first CT scan		0.001	1.00 (0.99–1.01)	0.37
Constant				0.000

^a^Adjusted for other variables in the model by logistic regression.

Nagelkerke pseudo *R*^2^ = 24.6%, Model *χ*^2^(12) = 41.1, *p* < 0.001.

AIS: Abbreviated Injury Scale; 95% CI: 95% confidence interval.

### Hospital LOS

Patients below the age of 60 and with AIS score >3 had a 4-fold lower median LOS than patients 60 years or older (*p* < 0.001, Table 2). There was no significant difference between the two age categories with respect to hospital LOS for AIS scores of 4 and 5. Age, triage priority, and AIS score of 5 were independent risk factors for hospital LOS in both age groups (Tables 3 and 4).

### Mortality

Mortality was higher in older patients for all AIS scores; however, this was only statistically significant for patients with AIS score of 3 (Table 5). Mortality was independently associated with age, triage priority, and AIS score, but not time from admission to first CT scan (Table 5).

## Discussion

Current literature indicates that there is under-triage of elderly trauma patients, including those with TBI, and that mortality in this group of patients is higher. Our present study suggests that elderly patients with TBI are under-triaged when using the Rapid Emergency Triage and Treatment System–Adult (RETTS-A). Thus, elderly patients less often receive the highest triage priority, despite similar AIS scores. Moreover, the time from ED admission to CT scan was longer for elderly patients with isolated TBI AIS scores of 3 and 4. While mortality was independently associated with age, triage priority, and AIS score, mortality was not associated with time from ED admission to first CT scan.

There is no widely accepted threshold or cut-off value that constitutes a ‘geriatric’ patient, nor is there a definite cut-off that accurately predicts outcome. Various age cut-off values have been proposed, ranging between 45 and 80 years, where most data show that trauma patients over 60 years have an increased mortality compared with younger patients (6). We therefore chose 60 years of age as our cut-off

It has been shown that for a given anatomical severity of TBI, elderly patients may present with a higher Glasgow Coma Scale (GCS) than younger patients (8). The reason for this could partly be explained by the fact that the brain undergoes age-related atrophy, allowing a greater haematoma size and more pronounced oedema before clinical signs are recognised (11). This may lead to under-triage of elderly patients and could also explain the poor performance of triage tools in elderly trauma patients (8).

TBI in older adults is associated with worse outcome when compared to younger adults (2). Our data are in accordance with these findings. We are not able to explain the effect of hospital on LOS; however, it is likely that the variation in LOS is dependent on local routines controlling the care of patients with TBI in the different wards included. Increased AIS score is most often related to affected levels of consciousness in both young and old patients, and triage priority increases with higher AIS score (12). However, even though AIS score and triage priority are related, they both uniquely contribute to the prediction of mortality. Similar to the findings of Kehoe et al., we also observed that around 50% of patients with an AIS score of 3 in both age groups were relatively unaffected by existing structural damage according to the triage priority (low triage priority) (8). However, we also observed a tendency that elderly patients with AIS scores >3 were more seldom triaged with the highest triage priority. On the contrary, we observed that elderly patients were more often triaged with the lowest priority. Furthermore, elderly patients had a significantly longer time from admission to first CT scan compared with younger patients with AIS scores of 3 and 4. This difference in trauma management could be explained by the inability of the current triage guidelines to detect serious intracranial injury in elderly patients related to a less affected level of consciousness (8).

Elderly patients have a higher incidence of medical comorbidities, tend to use anticoagulant medication more often than younger patients, and have lower physiological reserves, increasing their susceptibility to even minor trauma and disease (6). However, underlying medication and comorbidities are not considered in most triage tools, including RETTS-A (13). Similarly, age is not included as a core variable in RETTS-A. Among the most commonly used triage systems, only the Emergency Severity Index has been validated in patients >65 years. Most other systems have not been tested (14), and triage priority-related mortality is not adjusted for age or gender (13). Interestingly, a recent study has shown that age is identified as an independent risk factor for 1- and 30-day mortality in patients triaged according to RETTS-A. This association was more pronounced in patients with lower medical priority (lower triage priority groups) (7,15). Compared with patients <50 years of age, patients >80 years had an almost 50-fold increased risk of both 1- and 30-day mortality. In that study, age and level of consciousness were the strongest predictors for mortality (7). ED triage tools, including RETTS-A, rely heavily on vital signs upon arrival. Vital sign cut-offs are notoriously unreliable for elderly patients, which may affect the validity of RETTS-A and similar triage systems in elderly patients (16). Taken together, elderly trauma patients are at significant risk of under-triage using RETTS-A, because of multiple illness and polypharmacy, resulting in delays or incorrect management.

Older age may be an overly simplistic measure to understand outcomes in geriatric patients, and a better predictor may be the degree of frailty. The most commonly used definition of frailty is the physical frailty phenotype described by Fried et al., which is based on criteria related to reduced physical reserves (weight loss, exhaustion, weakness, slowness, and reduced physical activity) (17). However, the use of this definition in elderly trauma patients with affected consciousness might be difficult. Therefore, there is a requirement for other instruments to quantify frailty in elderly trauma patients, for example, the Trauma Specific Frailty Index (TSFI) (18). The lack of frailty measurement in the current triage system may explain the differences seen in this study.

It is well documented that elderly patients with hip fractures benefit from swift acute management in terms of length of hospital stay and mortality, and this is true also for patients 60 years or older with significant comorbidity (19). The present study shows that elderly patients with TBI are not prioritised in an acute setting, wait longer for their initial CT scan and have a longer LOS and higher mortality. Considering recent data showing that aggressive and early treatment of TBI in the aged population may be just as beneficial as in younger patients (4), it seems reasonable to focus more on this patient group.

Our study has some limitations. Importantly, only patients with isolated TBI without any other trauma were included. We did not have access to all relevant underlying diagnoses, medication, and comorbidities, as well as decisions regarding withdrawal of care, of the included patients. Another limitation is the low number of patients included in the study and its retrospective design.

In conclusion, we found that elderly patients with isolated TBI are managed differently than younger patients. The reason for our finding is unclear, and further studies are warranted to study triage according to RETTS-A in elderly patients.
